# A cost-effectiveness analysis of surgical care delivery in Eastern Uganda-a societal perspective

**DOI:** 10.1186/s12913-023-09216-x

**Published:** 2023-03-15

**Authors:** Obieze Nwanna–Nzewunwa, Esther Agwang, Melissa Carvalho, Mary-Margaret Ajiko, Rasheedat Oke, Christopher Yoon, Mohamed M Diab, Fred Kirya, Elliot Marseille, Catherine Juillard, Rochelle A. Dicker

**Affiliations:** 1 Department of Surgery, Maine Medical Center, 22 Bramhall Street, Portland, ME 04102 USA; 2 Department of Surgery, Soroti Regional Referral Hospital, Soroti, Uganda; 3 Program for the Advancement of Surgical Equity (PASE), University of California Los Angeles, Los Angeles, CA USA; 4 David Geffen School of Medicine, University of California Los Angeles, Los Angeles, CA USA; 5 Department of Surgery, Duke University Medical Center, Durham, NC USA; 6 Principal, Health Strategies International, 555 59th Street, Oakland, CA USA

**Keywords:** Cost-effectiveness, QALY, Femur fractures, Surgical care cost, Societal cost, Uganda, Africa, Epidemiology, Global Surgery

## Abstract

**Background:**

The mismatch between the global burden of surgical disease and global health funding for surgical illness exacerbates disparities in surgical care access worldwide. Amidst competing priorities, governments need to rationally allocate scarce resources to address local needs. To build an investment case for surgery, economic data on surgical care delivery is needed. This study focuses on femur fractures.

**Methods:**

This prospective cohort study at Soroti Regional Referral Hospital (SRRH), captured demographic, clinical, and cost data from all surgical inpatients and their caregivers at SRRH from February 2018 through July 2019. We performed descriptive and inferential analyses. We estimated the cost effectiveness of intramedullary nailing relative to traction for femur fractures by using primary data and making extrapolations using regional data.

**Results:**

Among the 546 patients, 111 (20.3%) had femur fractures and their median [IQR] length of hospitalization was 27 days [14, 36 days]. The total societal cost and Quality Adjusted Life Year (QALY) gained was USD 61,748.10 and 78.81 for femur traction and USD 23,809 and 85.47 for intramedullary nailing. Intramedullary nailing was dominant over traction of femur fractures with an Incremental Cost Effectiveness Ratio of USD 5,681.75 per QALY gained.

**Conclusion:**

Femur fractures are the most prevalent and most expensive surgical condition at SRRH. Relative to intramedullary nailing, the use of femur traction at SRRH is not cost effective. There is a need to explore and adopt more cost-effective approaches like internal fixation.

**Supplementary Information:**

The online version contains supplementary material available at 10.1186/s12913-023-09216-x.

## Background

The global burden of surgical disease is profound and disproportionately affects low- and middle-income countries (LMICs). Basic lifesaving surgery is unavailable to over 70% of the world’s population [1, 2]. Disparity in surgical care access is further accentuated by unequal allocation of global health funding and resources. While global health priorities like HIV (23%), child health (21%), maternal health (12%), malaria (6%) and tuberculosis (4%) received a significant proportion of the $41 billion total global health funds allocated in 2019, noncommunicable diseases, which encompasses surgical conditions like trauma, remain underfunded (1.6% of total global health funds allocated in 2019) and underprioritized despite accounting for a larger global disease burden [3, 4]. This funding mismatch reflects historic misconceptions that surgical care delivery is expensive and unscalable and not a component of Universal Health Coverage (UHC).

Over the past two decades, a series of events and research have elucidated the magnitude of the global surgical disease burden as well as the importance of surgical care access and delivery in global public health [5–8]. In 2015, the World Health Assembly formally recognized the role of surgical care access as a crucial component of UHC [9]. This catalyzed concerted stakeholder efforts and government engagement to prioritize surgical conditions and their care. Subsequently, some LMICs developed National Surgical, Obstetric, and Anesthesia Plans (NSOAPs), which include roadmaps to facilitate the prioritization of surgical care delivery within their national strategic health plans and policies [10].

Investing in essential surgical care delivery in LMICs is cost-effective and projected to save about 12.3 trillion dollars between 2015 and 2030 [2]. The World Bank recommends early financing of surgical care access for any country seeking to achieve UHC [5]. LMIC governments are required to objectively allocate scarce resources to meet the needs of their population. Such key decisions should be data-driven, however, data on the epidemiology and economics of surgery in LMICs remains limited.

Cost effectiveness analyses are analytical methods used to compare alternative interventions in a consistent framework to ensure selection of the intervention representing the most efficient use of limited resources [11, 12]. Thus, cost effectiveness analyses of surgical care delivery in LMICs is essential to identifying key surgical conditions and interventions that merit prioritization by stakeholders. Accurate and context-appropriate cost effectiveness analysis can improve surgical access and quality by providing evidence and highlighting optimal interventions that will inform NSOAPs and national health agendas.

Prior studies suggest that surgical care at the district level in Africa is highly cost effective [5, 13]. However, such studies are lacking from rural Eastern Uganda. Thus, in this study, we sought to ascertain the cost-effectiveness of femur fracture surgical management delivery in rural Uganda. In prior studies, we showed the high incidence and cost of femur fractures in Soroti Regional Referral Hospital (SRRH) [14, 15]. The current available management option for femur fractures at SRRH is traction immobilization which has a lengthier hospital length of stay and poorer clinical outcomes than internal fixation methods such as intramedullary (IM) nailing [14]. This study describes the epidemiology, surgical management options, effectiveness of femur fracture management, and the outcomes, costs, and cost-effectiveness of surgical care delivery at SRRH, a rural district-level regional referral hospital in Uganda. We hypothesize that at SRRH, the current practice of femur fracture management with traction is not cost effective.

## Methods

This cost effectiveness analysis is part of a larger study aimed at evaluating the economics of surgical care access in Soroti, Uganda and part of the methodology has been previously described and will be referenced appropriately in this study [14].

### Study setting, subjects, and enrollment

As previously described [15], Soroti Regional Referral Hospital (SRRH) is a 300-bed public hospital in Eastern Uganda. We enrolled all adult patients and their caregivers who were admitted to SRRH with a surgical condition between February 2018 and January 2019.

### Study instruments

Three data collection instruments were used in this study. Instruments 1 and 2 were novel and were devised for the purpose of this study. The third instrument, the EQ-5D-5L-VAS questionnaire [16], was used to characterize the health state of patients.

#### Instrument 1: patient data collection form

This instrument collected patients’ sociodemographic (age, sex, district, occupation, family size, role in the family), clinical (e.g. diagnosis, length of stay, medications and treatment received), and cost (e.g. transportation, medications, and investigations procured outside SSRH) data. In this study, cost is described from the societal perspective. “Societal cost” encompasses all costs incurred by all parties (patients, their caregivers, the hospital, the government, and any other entity involved) towards the patient obtaining surgical care [14, 17, 18].

#### Instrument 2: caregiver data collection form

This instrument focused on the patients’ caregivers and elicited sociodemographic data (e.g., age, sex, occupation etc.) and cost data (e.g., their transportation, lost wages etc.) related to the patient’s hospitalization.

#### Instrument 3: EQ5D-5L-VAS

The EQ-5D-5L-VAS is a standardized questionnaire that is used to characterize and value the health state of patients, thereby making it heterogenous disease entities comparable in order to facilitate a cost-effectiveness analysis [16]. It assesses 5 dimensions of health (mobility, self-care, usual activities, pain/discomfort and anxiety/depression), each on a 5-point Likert scale. It also comprises a visual analogue scale (VAS) measured from 0–100 with which patients are asked to rate their state of health. Due to perceived metric literacy challenges, a modification was made to the VAS by drawing a bottle over the VAS and asking the participants to rate how “full” they felt their health status was, using the level of fullness of the bottle as a reflection of their health status and the corresponding level on the VAS was then obtained.

### Data collection

There were two phases of primary data collection: 1) the inpatient phase, and 2) the outpatient phase.

#### Inpatient phase

From February 2018 through January 2019, we enrolled all consented participants into the study and through a combination of direct observation of the processes of care and interviews, we obtained patient-related demographic, social, clinical, and cost data using data collection instrument 1 (Additional file 1). We also obtained sociodemographic and cost data from caregivers using instrument 2 (Additional file 2). The EQ-5D-5L- VAS questionnaire was administered on admission and at discharge.

#### Outpatient phase

After discharge, patients were given a follow up phone call 3 weeks, 3 months, and 6 months from the date of discharge. During each follow up call the researcher inquired about the patients’ health state, complications, medical expenses related to their hospitalization, and the EQ-5D-5L questionnaire was administered.

Interviews were conducted in English, Ateso, Swahili, or Luganda by a local researcher who was fluent in these languages and translated non-English interviews to English. All data were collected on paper, then entered into Microsoft Excel [19] and then analyzed.

#### Economic data inputs and sources

In accordance with the recommendation of the Second Panel for Cost Effectiveness in Medicine, our Impact Inventory Template (Table 1) details the various health outcomes and cost inputs used in this study [20]. Using instrument 1, we obtained primary data about patients’ direct expenditures (transportation, medications and investigations procured outside SRRH) from patients and caregivers. Caregiver expenditures were similarly obtained using instrument 2. Per government policy, healthcare in Ugandan public hospitals is free of charge. Thus, patients did not directly incur a medical bill for accommodation, medication, or surgical care. Such expenses were elicited from the hospital and government perspective and these cost inputs are also described (Table 1). The cost of surgical procedures was obtained by interviewing one of the three local surgeons at SRRH with prior experience as the hospital administrator at SRRH and extensive knowledge of the cost of surgical procedures in the public and private sector of healthcare in Soroti. These cost estimates were reviewed and endorsed by a second surgeon at SRRH. We also interviewed an orthopedic officer well versed in the cost, equipment, supplies and services required to deliver femur fracture care at SRRH. The cost of hospital staff, utilities, and accommodation were obtained from secondary sources. Cost data were obtained in Ugandan shillings (USh) and converted to United States Dollar (USD) using the prevalent World Bank 2018 exchange rate (USh 3,727 per dollar) [21]. We used data from the Ugandan 2017 national survey income database as the source of wages for the various job description in Soroti. Then we conservatively adjusted for inflation to derive the real wages in 2018 to reflect the occupation distribution of the subjects. This was done by using the formula Real wage = W/i, where W was the wage obtained from the 2017 survey and i = 2018 inflation rate (which was 2.55% per Statista.com).

**Table 1:** Impact inventory table with sources of cost inputs

Sector	Type of impact	Included in this reference case analysis from the societal perspective		Sources of evidence
		Health sector	Societal	
Formal health sector				
Health	Health outcomes (effects)			
	Longevity effects	Yes		Primary data from Instrument 1
	Health-related quality of life effects	Yes		Primary data from Instrument 1
	Complications	Yes		Primary data from Instrument 1
	Medical costs			
	Patients’ direct cost (drugs, tests, and supplies)		Yes	Primary data from Instrument 1
	Government’s cost (patient drugs, tests, and supplies)		Yes	Hospital inventory
	Cost of surgical procedure		Yes	Primary data from local providers
	Caregivers’ direct costs		Yes	Primary data from Instrument 2
	Hospital accommodation cost		Yes	World Health Organization WHO-CHOICE database [2]
	Staff cost		Yes	Bellamkonda N, et al. [3]
	Ancillary and administrative		Yes	Bellamkonda N, et al. [3]
Informal Health Care sector				
Health	Patient time		Yes	Primary data from Instrument 1
	Unpaid caregiver-time cost		Yes	Primary data from Instrument 1
	Patients’ and caregivers’ transportation		Yes	Primary data from Instrument 1
Non-Health Care Sector				
Productivity	Lost patient wages		Yes	Uganda National Household Survey [1]
	Lost caregiver wages		Yes	Uganda National Household Survey [1]
Education	Impact of hospitalization on the population’s education		NA	
Utilities	Water and sewerage		Yes	Uganda National Water and Sewerage Corporation [4]
Other	Exchange		Yes	World Bank Database [5]

### Data analysis

#### Quantitative data

We subjected the sociodemographic, clinical, and economic data to descriptive statistical analysis and expressed results as frequencies, medians, and proportions.

#### Cost effectiveness analysis

The societal cost of treatment of each surgical condition was obtained by adding all costs incurred to provide that treatment from the perspective of the patient, their attendant(s), the hospital, and government. Data from the EQ-5D-5L questionnaire were used to calculate the quality adjusted life years (QALYs) gained by treating each surgical condition. The QALYs gained by each patient was calculated using the formula:

$$\mathrm{QALY}=\mathrm{Years}\;\mathrm{of}\;\mathrm{Life}\;\mathrm{Lost}\;\mathrm{due}\;\mathrm{to}\;\mathrm{disability}\times\mathrm{Utility}\;\mathrm{value}$$

The years of life lost due to disability (YLL) was captured using instrument 1. To determine the utility value, the EQ-5D-5L questionnaire was used [16]. Per protocol, the EQ-5D-5L responses were aggregated to form a 5-digit code, which was then converted to a health utility value using the EQ-5D-5L “value set” to reflect how good or bad a health state is according to the preferences of the region. The Ethiopian value set was used for this study as it was the value set that most closely represents the Ugandan culture and valuation of health [22]. The QALYs were determined at admission, discharge, and 6 months, with the 6-month QALY representing the long-term outcome.

We determined the Cost Effectiveness Ratio (CER) of treating each surgical condition encountered at SRRH, by dividing the societal cost of treating a given surgical condition by the QALYs gained after treating that condition.

$$\mathrm{Cost}\;\mathrm{Effectiveness}\;\mathrm{Ratio}\;(\mathrm{CER})=\mathrm{Societal}\;\mathrm{cost}\;\mathrm{of}\;\mathrm{treatment}/\mathrm{QALYs}\;\mathrm{gained}\;\mathrm{after}\;\mathrm{treatment}$$

Lastly, we determined the Incremental Cost Effectiveness Ratio (ICER) of femur fracture treatment with traction compared against intramedullary nailing using the formula below.

$$ICER=\frac{\left[\mathrm{Cost}\;\mathrm{of}\;\mathrm{femur}\;\mathrm{traction}-\mathrm{cost}\;\mathrm{of}\;\mathrm{femur}\;\mathrm{nailing}\right]}{\left[\mathrm{QALYs}\;\mathrm{traction}-\mathrm{QALYs}\;\mathrm{nailing}\right]}$$

Since no patient at SRRH received IM nailing, primary data on the costs, QALYs and other outcomes of IM nailing were unavailable. We estimated the cost, QALYs and outcomes of IM nailing using regional data and the following assumptions:

##### Assumption 1

The median length of stay (LOS) for femur nailing patients and their attendants is 7 days. Data from Uganda shows the typical LOS for femur nailing patients to be 6.9 days after treatment [23]. Based on this, each patient’s and their attendants’ hospitalization cost (LOS x average patients daily cost) and the lost wages (LOS x daily income) were calculated.

##### Assumption 2

The QALYs gained by treating femur fractures in the Malawian population are similar to that of the neighboring Ugandan population. The literature suggests that patients who receive IM nailing have lower incidence of nonunion, malunion, orthostatic pneumonia, and length of stay of stay compared to traction [24, 25]. There was limited data from Uganda on the QALYs gained from skeletal traction and intramedullary nailing of femur fractures. So, we obtained data from Malawi and extrapolated the QALY data to the population at SRRH and used it to perform the ICER calculation.

##### Assumption 3

Patients lost to mobile phone follow up at 6 months have similar QALYs as those captured via mobile phone follow-up. To account for missing follow-up outcomes (QALY) data, we extrapolated the mobile phone follow-up outcome data obtained from patients who were accessible via mobile phone, to account for patients who could not be reached via mobile phone.

#### Sensitivity analysis

Given that we previously established that the length of stay was the key driver of cost of surgical care [14], we conducted a sensitivity analysis using the length of stay as the independent variable of interest. We selected the 25^th^ and 75^th^ percentile levels of the length of stay (which correspond to the interquartile range for length of hospitalization) and determined the ICERs at both levels to ascertain if IM nailing was cost effective at those levels.

## Results

We enrolled 99.6% (546) of the 548 patients that were admitted to the SRRH surgical ward with a surgical ailment during the first 11 months (inpatient phase of the study). The patients were predominantly males (62%) at an average age of 30 years (SD ± 28.8) who identified as being single (53%) or married (40%) with primary (55%) or secondary (11%) education as their highest level of education (Table 2). These patients were chiefly peasant farmers (42%) and students (31%). Patients with access to a mobile phone (59%) were followed up via phone call until 6 months after their discharge (long term follow up).

**Table 2:** Demography of patients (*n* = 546) and caregivers (*n* = 615)

Variables	Patients (*n* = 546)	Patients' caregiver (*n* = 615)
Age, median [IQR]	22 [7, 49]	35, [28, 45]
**Sex**	**(*n* = 546)**	**(*n* = 601)**
Male	340 (62.27%)	187 (31.1%)
Female	206 (37.73%)	414 (68.9%)
**Marital status**	**(*n* = 544)**	**(*n* = 601)**
single	289 (53.1%)	40 (6.7%)
married	215 (39.5%)	523 (87.0%)
divorced	0	3 (0.5%)
widowed	40 (7.35%)	35 (5.8%)
**District**	**(*n* = 545)**	**(*n* = 601)**
Amuria	106 (19.45%)	117 (19.4%)
Kabermaido	26 (4.77%)	29 (4.8%)
Katakwi	33 (6.06%)	37 (6.2%)
Ngora	27 (4.95%)	26 (4.3%)
Palisa	1 (0.18%)	1 (0.2%)
Serere	71 (13.03%)	77 (12.8%)
Soroti	265 (48.62%)	287 (47.8%)
Bukedea	5 (0.92%)	5 (0.8%)
Buyende	1 (0.18%)	1 (0.2%)
Other	10 (1.83%)	21 (3.5%)
**Education**	**(*n* = 546)**	**(*n* = 600)**
None	76 (13.92%)	61 (10.1%)
Pre-primary	31 (5.68%)	0 (0%)
Primary	301 (55.13%)	443 (73.5%)
Secondary	58 (10.62%)	76 (12.6%)
Tertiary	11 (2.01%)	20 (3.3%)
N/A	69 (12.64%)	0 (0%)
**Occupation**	**(*n* = 546)**	**(*n* = 599)**
Peasant Farmer	230 (42.12%)	481 (80.3%)
Student	169 (30.95%)	21 (3.5%)
Business owner	11 (2.01%)	33 (5.5%)
Driver	10 (1.83%)	12 (2.0%)
Teacher	8 (1.47%)	6 (1.0%)
Housewife	7 (1.28%)	16 (2.7%)
Retired	2 (0.37%)	1 (0.2%)
Other	21 (3.80%)	29 (4.8%)
Underaged for work/school	88 (16.12%)	0 (0%)
**Other socioeconomic indices**	**(*n* = 546)**	**(*n* = 615)**
Household owns a Cellphone	313 (59.06%)	230 (39.1%)
I am the breadwinner	298 (54.58%)	191 (31.9%)
Number of dependents (mean ± SD)	7.3 ± 4.0	7.0 ± 4.6

**Fig. 1:**
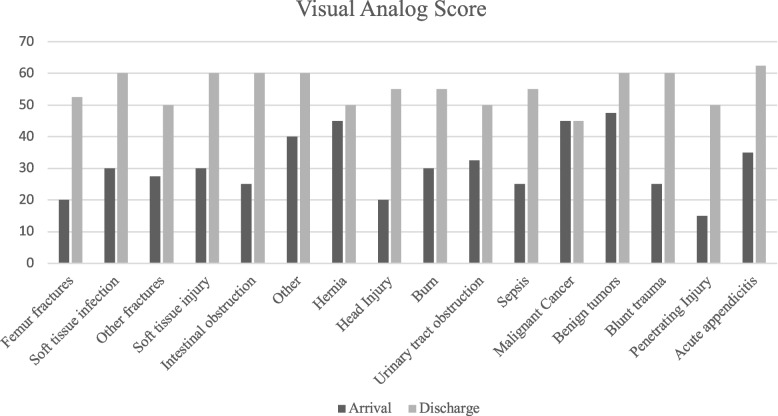
Comparing the median Visual Analogue Scale (VAS) score on admission and discharge

The most prevalent surgical condition encountered were femur fractures (20.3%) followed by soft tissue infections (12.3%), non-femur fractures (i.e. all fractures other than femur fractures) (11.9%), soft tissue injuries (10.7%), and intestinal obstructions (7.7%). The median [IQR] length of stay for all patients was 7 days [3,17]. The median [IQR] length of stay for patients with femur fractures (27 days [14, 36 days]) was significantly longer than all other surgical conditions intestinal obstructions (9 days [7,12]), soft tissue infections (8 days [3,15]), non-femur fractures (4 days [2, 8]), hernias (4 days [2.5,5.5]), and soft tissue injuries (3 days [2,6.5]) (*p* = 0.0001). There was an improvement in the self-reported health status in all surgical disease groups (Fig. 1). Patients with penetrating injuries had the largest improvement (+ 35 points) in self-reported health status, with a median VAS at discharge of 50 [50,55] compared to a median VAS of 15 [IQR 15,20] at the time of admission. Conversely, patients with cancer had the lowest median VAS at discharge (45 [IQR 30,55] and the least improvement (Median 0 [-2.5, 5]) in self-reported health status. Patients with appendicitis experienced the highest self-reported heath status at discharge (Median VAS of 62.5 [50,70]). A total of 69 inpatient complications (14%) were identified (Table 3). Malaria (5.6%) death (2.7%), sepsis (1.8%) and pneumonia (1.4%) were most prevalent complications. Other notable complications were peritonitis, wound infection, malunion and osteomyelitis.

**Table 3:** Inpatient complications of surgical conditions

Diagnosis	Death		Pneumonia		Malaria		Sepsis		Peritonitis		Wound sepsis		Malunion		Osteomyelitis		Total, % (*n* = 504)	
Femur fractures (*n* = 103)	4	4%	2	2%	9	9%	1	1%	1	1%	3	3%	1	1%	1	1%	22	4.3%
Soft tissue infection (*n* = 62)	-	-	-	-	5	8%	3	5%	-	-	1	2%	-	-	-	-	9	1.8%
Other fractures (*n* = 60)	-	-	2	3%	2	3%	-	-	-	-	-	-	-	-	-	-	4	0.8%
Soft tissue injury (*n* = 54)	-	-	-	-	4	7%	1	2%	-	-	-	-	-	-	-	-	5	1%
Intestinal obstruction (*n* = 39)	3	8%	1	3%	5	15%	1	3%	4	10%	-	-	-	-	-	-	14	2.8%
Other (*n* = 33)	3	9%	1	3%	1	3%	1	3%	-	-	-	-	-	-	-	-	6	1.2%
Hernia (*n* = 29)	-	-	-	-	-	-	-	-	-	-	-	-	-	-	-	-	0	0%
Head Injury (*n* = 27)	1	4%	1	4%	1	4%	-	-	-	-	-	-	-	-	-	-	3	0.6%
Burn (*n* = 26)	-	-	-		1	4%	-	-	-	-	-	-	-	-	-	-	1	0.2%
Urinary tract obstruction (*n* = 16)	-	-	-	-	-	-	2	13%	-	-	-	-	-	-	-	-	2	0.4%
Sepsis (*n* = 15)	2	13%	-	-	-	-	-	-	-	-	-	-	-	-	-	-	2	0.4%
Malignant Cancer (*n* = 13)	1	8%	-	-	-	-	-	-	-	-	-	-	-	-	-	-	1	0.2%

Over half (55%) of the patients had at least one individual that depended on them financially for sustenance. Patients reported a median of 7 [IQR = 4, 9] dependents. Patients were often accompanied by caregivers ranging from 0–5 in number. We enrolled all caregivers (*n* = 615) that we encountered. The caregivers were predominantly married (87%) women (69%), at an average age of 37.7 years (SD ± 12.7) years with a primary education (74%) as their highest level of education. Fifty-one percent of patients (*n* = 546) and 81% of caregivers (*n* = 550) reported having an occupation that yielded income. The average lost wages attributable to hospitalization for surgical disease was USD56 for patients and USD22 for caregivers. The total lost wages incurred was USD15,781 for patients, USD12,051 for caregivers and USD 27,832 for the whole household.

The total societal cost of femur traction for the 111 femur fracture patients was USD 61,748.10. The societal cost of intramedullary nailing for the same patients was estimated to USD 23,907.64. Data from Malawi showed the QALYs gained by treating each patient with traction was 0.71 as opposed to 0.77 for intramedullary nailing [26]. For the study population, the QALYs gained by treating 111 patients with traction is estimated as 78.81 as opposed to 85.47 for intramedullary nailing.

$$ICER=\frac{\left[\mathrm{Cost}\;\mathrm{of}\;\mathrm{femur}\;\mathrm{traction}-\mathrm{cost}\;\mathrm{of}\;\mathrm{femur}\;\mathrm{nailing}\right]}{\left[\mathrm{QALYs}\;\mathrm{traction}-\mathrm{QALYs}\;\mathrm{nailing}\right]}$$

$$ICER=\frac{\left[\mathrm U\mathrm S\mathrm D\;61,748.10-\mathrm U\mathrm S\mathrm D\;23,907.64\right]}{\left[78.81-85.47\right]}$$

$$\mathrm{ICER}=-\mathrm U\mathrm S\mathrm D\;5,681.75\mathrm p\mathrm e\mathrm r\;\mathrm Q\mathrm A\mathrm L\mathrm Y\;\mathrm g\mathrm a\mathrm i\mathrm n\mathrm e\mathrm d$$

The estimated ICER for treating 111 femur fracture patients with traction intramedullary nailing compared to skeletal traction was – USD 5,681.75 per QALY gained).

### Sensitivity analysis

We used a minimum and maximum length of stay corresponding to the 25^th^ and 75^th^ percentiles for patients’ length of stay, we estimated the ICER to be—USD 3,452.50 at the 25^th^ percentile of length of hospitalization and -USD 10,820.77 at the 75^th^ percentile level of the length of hospitalization.

## Discussion

This study provides valuable information that makes stakeholders better able to compare the value and benefits obtained by employing various surgical interventions. It highlights the key needs and improvement opportunities of the rural surgical health system and the foundation for making an investment case for the use of internal fixation and open fracture management techniques in the management of femur fractures. Femur fractures were the most prevalent and expensive surgical conditions encountered at SRRH. Our findings show that most surgical patients were young and in their most productive decades of life. The societal cost of treating surgical conditions is profound and chiefly driven by the length of hospitalization [14, 27].

The use of traction for femur fracture treatment has a hidden cost, which is the opportunity cost of lost wages or education or both. In the African setting, this cost is borne by the patient and their household and is more apparent from a societal perspective. Length of hospitalization drives the societal cost of surgical care at SRRH [14, 25, 28]. Interventions associated with shorter hospitalization (e.g. internal fixation) may lower costs, but will require fiscal commitment from the Ugandan government, which may be a disincentive compared to traction that currently does not require any capital investment for its continued use. Data from similar setting suggest that internal fixation techniques in this setting will be cost saving [24, 29]. In order to prioritize more valuable healthcare interventions for the same dollar spent, governments must look beyond healthcare costs in isolation and identify cost effectiveness of health interventions.

Our findings suggest that adopting intramedullary nailing may offer superior clinical benefits to patients, as well as superior financial benefits to the society. For every QALY gained when treating femur fractures, there is a potential cost saving of USD 5,681.75 if the intramedullary nailing technique is used as opposed to traction. Based on our sensitivity analysis, we found intramedullary nailing to be cost saving relative to traction with an ICER ranging from—USD 3,452.50 (at the 25th percentile of length of stay) and -USD 10,820.77 (at the 75^th^ percentiles for length of hospitalization). Thus, relative to intramedullary nailing, the use of traction in the treatment of femur fractures at SRRH is not cost effective relative from a societal perspective. Similar studies conducted in similar settings have proven intramedullary nailing to be cheaper and more cost effective than traction [24, 29]. The shorter length of hospitalization for both patients and their caregivers is a key component of the observed cost benefit [14]. There are also better outcomes with the use of open fracture treatment techniques [23, 26, 30, 31]. Currently, SRRH lacks the resources (e.g. external fixators, internal fixators, intramedullary nails) required to perform open femur fracture treatment [14, 28]. In view of above, we recommend that the government and stakeholders in Uganda further explore and strongly consider the use of intramedullary nailing techniques in the management of femur fractures. Less expensive femur ORIF options with good outcomes have been reported in LMIC settings [23].

The overall inpatient complication rate among all patients was low (14%) and the inpatient mortality rate was 3%. Femur fracture patients had 22% complication rate, second to patients with intestinal obstruction (39%). A key consideration is that femur fracture patients had a longer in hospital stay and, thus, they were under direct clinical observation for much longer than all other patient groups. Therefore, there is a higher likelihood that a complication, if it did occur, would be captured in a patient with a longer in hospital stay due to the longer period of direct clinical supervision as opposed to discharge patients.

The opportunity cost attributable to surgical disease includes but is not limited to lost wages and missed education. In Africa, this cost is borne by patients, and their caregivers. Caregivers have become an informal, but essential component of the African health system. They perform essential functions the would traditionally be performed by nursing staff, mental health providers, social workers, physical therapist etc [32, 33].. Despite free care in Uganda, the need to access surgical care continually threatens to impoverish patients and their households [14, 34]. Approximately 41% of Ugandans live below the international poverty line of USD1.90 per day; this is higher in Soroti district (51%) and Eastern Uganda (84%) in general [35, 36]. Caregivers at SRRH lost an average of USD 22 over a median hospital stay of 7 days. This is a relatively high cost and raises concern for possible medical impoverishment due to lost caregiver wages. It is a perspective that has not been previously appreciated or appropriately characterized and it is an opportunity for future research.

Patient follow-up can be challenging in developing settings for reasons not limited to financial, sociocultural, geographic, healthcare access issues and low use of electronic medical records. Tracking patients after discharge is arduous, hence our approach of using mobile phone follow up which had certain merits and demerits. Mobile follow up was well received by patients. An unintended benefit was that patients reported feeling more valued, as most had never experienced a follow up phone call after their prior hospital visits. They voiced feelings of trust towards SRRH and their providers. Some patients did not have mobile phone access, and this limited our ability to access and assess their outcomes. Mobile phone access can be used as a surrogate for socioeconomic status in developing settings, with the lack of mobile phone ownership indicating a lower socioeconomic status. Although the follow up rates in this study (59%) closely approaches that in the literature (60–89%). It is possible the subjects that were not accessible via mobile phone are predominantly of a low socioeconomic demographic and would hypothetically have worse financial hardship and clinical outcomes.

### Limitations of the study

This study has some limitations. Open femur repair was unavailable at SRRH at the time of this study and publication, thus a prospective comparison of direct costs and outcomes was not feasible given the absence of an open femur fracture treatment arm. It is our hope that this study will inspire investment in open fracture treatment at which point, a prospective comparison of femur traction with open treatment of femur fracture would be feasible. Because healthcare at Ugandan government facilities is free, the prevailing sociocultural milieu and local stakeholders informed a decision not to ask participants directly about their income, so as not to create a perception that their income would influence their care. The use of Malawian data to determine the QALYs gained after IM nailing and traction of femur fractures assumes that both patient populations and treatment techniques are similar. While we do not have data that they are not, it is still very possible that they are dissimilar. Using secondary data from the government surveys to estimate wages, we made a conservative adjustment that may underestimate the income and lost wages due to hospitalization. The IM nailing and ICER estimates do not factor in the initial investment cost to set up IM nailing services, however, the authors believe the potential cost saving and benefits outweigh such initial investments. Also, the inability to monetize the cost of missed education further understates the economic impact of surgical conditions in general. This is particularly true for femur fractures and other conditions that predominantly affect younger and school aged patients.

Patient follow-up is a global healthcare challenge. Although our follow up rates (59% of all patients and 59% femur fracture patients) approximates other published rates (60–89%) in Africa, it is important to highlight that mobile phone follow up may preferentially highlight characteristics of patients who have access to a mobile phone, who typically may be of a higher socioeconomic status than subjects that lack access to a mobile phone. Another challenge is the limited capacity to perform a physical examination since it relies more on patients’ ability to recognize and describe complications [37–39]. It is plausible that complications may have been unrecognized in the inpatient period, but particularly after discharge. Also, it is possible that those lost to follow-up had more adverse outcomes or hardship. Lastly, in a study that follows up patients, there is a risk of recall bias. Patients who experienced an adverse event either inpatient or at any point during the 6-month follow up period may preferentially report worse outcomes (economic, clinical or QALY etc.) However, since all patients got the same treatment, it is less likely that recall bias would significantly skew towards either arm nor that it would affect the results of this cost-effective analysis.

## Conclusion

Compared with all other surgical conditions treated at SRRH, the use of traction in the management of femur fracture is expensive, associated with prolonged hospitalization, higher morbidity and mortality and from a societal perspective, it is ultimately not cost effective. From the societal perspective, this study reflects a more wholistic and realistic estimate of the cost and outcomes associated with surgical care delivery as it encompasses patient, caregiver, transportation and other non-health sector costs [20]. We recommend that stakeholders in Uganda explore the use of intramedullary nailing and open surgical fixation for the management of femur fractures as opposed to the use of traction.

## Supplementary Information

**Additional file 1.**

**Additional file 2.**

## Data Availability

The datasets used during the current study are available from the corresponding author on reasonable request.

## Abbreviations

LMICs: Low- and Middle-Income countries
UHC: Universal Health Coverage
NSOAPs: National Surgical Obstetric and Anesthesia Plans
SRRH: Soroti Regional Referral Hospital
IM: Intramedullary
VAS: Visual Analogue Scale
USh: Ugandan Shillings
USD: United States Dollar
QALYs: Quality Adjusted Life Years
YLL: Years of life lost due to disability
CER: Cost Effectiveness Ratio
ICER: Incremental Cost Effectiveness Ratio
LOS: Length of Stay
IQR: Interquartile Range

## References

[1] Mazumdar T. Five Billion People ‘Have No Access to Safe Surgery’- BBC News. BBC News. http://www.bbc.com/news/health-32452249. Published 2015. Accessed 21 August, 2015.

[2] Meara JG, Leather AJM, Hagander L (2015). Global Surgery 2030: Evidence and solutions for achieving health, welfare, and economic development. Surgery..

[3] Gutnik LA, Yamey G, Dare AJ (2015). Financial contribution to global surgery: an analysis of 160 international charitable organisations. Lancet (London, England)..

[4] Institute for Health Metrics and Evaluation (IHME). Financing Global Health 2019: Flows of global health financing. Seattle: IHME; 2019. https://vizhub.healthdata.org/fgh/.

[5] Debas HT, Donkor P, Gawande A, Jamison DT, Kruk ME, Mock CN. Disease Control Priorities, 3rd Edition: Volume 1: Essential Surgery; 2015. 10.1596/978-1-4648-0346-8 26740991

[6] Stewart B, Khanduri P, McCord C (2014). Global disease burden of conditions requiring emergency surgery. Br J Surg..

[7] Marseille E, Morshed S (2014). Essential surgery is cost effective in resource-poor countries. Lancet Glob Heal.

[8] Haagsma JA, Graetz N, Bolliger I (2013). The global burden of injury: incidence, mortality, disability-adjusted life years and time trends from the Global Burden of Disease study. Inj Prev.

[9] Price R, Makasa E, Hollands M (2015). World Health Assembly Resolution WHA68.15: “strengthening Emergency and Essential Surgical Care and Anesthesia as a Component of Universal Health Coverage” - Addressing the Public Health Gaps Arising from Lack of Safe, Affordable and Accessible Surgical a. World J Surg.

[10] Peters AW, Roa L, Rwamasirabo E (2020). National surgical, obstetric, and anesthesia plans supporting the vision of universal health coverage. Glob Heal Sci Pract.

[11] Sanders GD, Maciejewski ML, Basu A (2019). Overview of Cost-effectiveness Analysis. JAMA.

[12] Sanders GD, Neumann PJ, Basu A (2016). Recommendations for conduct, methodological practices, and reporting of cost-effectiveness analyses: second panel on cost-effectiveness in health and medicine. JAMA.

[13] Bentounsi Z, Sheik-Ali S, Drury G, Lavy C (2021). Surgical care in district hospitals in sub-Saharan Africa: a scoping review. BMJ Open.

[14] Nwanna-Nzewunwa O, Oke R, Agwang E (2021). The societal cost and economic impact of surgical care on patients’ households in rural Uganda; a mixed method study. BMC Health Serv Res..

[15] Nwanna-Nzewunwa OC, Ajiko MM, Kirya F (2016). Barriers and facilitators of surgical care in rural Uganda: A mixed methods study. J Surg Res..

[16] EuroQol Research Foundation. EQ-5D-5L User Guide. https://euroqol.org/publications/user-guides. Published 2019. Accessed Dec 1, 2021.

[17] Chapel J. Economic evaluation: intervention cost analysis in Public Health. In: CDC Coffee Break. Atlanta; 2012. https://www.cdc.gov/dhdsp/docs/CB-July2018-508.pdf.

[18] Fakhri MA, Juni MH, Faisal I (2017). Societal perspective in economic evaluation. Int J Public Heal Clin Sci..

[19] Microsoft Corporation. Microsoft Excel 2011 for Mac version 14.3.6. Redmond: Microsoft Corporation; 2011.

[20] Sanders GD, Neumann PJ, Basu A (2016). Recommendations for conduct, methodological practices, and reporting of cost-effectiveness analyses: Second panel on cost-effectiveness in health and medicine. JAMA - J Am Med Assoc.

[21] World Bank Group. Official exchange rate (LCU per US$, period average) - Uganda. https://data.worldbank.org/indicator/PA.NUS.FCRF?name_desc=false&locations=UG. Published 2020. Accessed 19 Sept 2020.

[22] Welie AG, Gebretekle GB (2020). Valuing Health State: an EQ-5D-5L value set for Ethiopians. Value Heal Reg Issues.

[23] Sekimpi P, Okike K, Zirkle L, Jawa A (2011). Femoral fracture fixation in developing countries: an evaluation of the Surgical Implant Generation Network (SIGN) Intramedullary Nail, The Journal of Bone & Joint Surgery. J Bone Jt Surg..

[24] Parkes RJ, Parkes G, James K (2017). A systematic review of cost-effectiveness, comparing traction to intramedullary nailing of femoral shaft fractures, in the less economically developed context. BMJ Glob Heal..

[25] Kamau DM, Surg MO, Gakuu LN, Surg M, Ecsa FCS (2014). Comparison of closed femur fracture: Skeletal traction and intramedullary nailing cost-effectiveness. East African Orthop J.

[26] Chokotho L, Donnelley C, Young S, Lau BC, Wu HH, Mkandawire N, Gjertsen JE, Hallan G, Agarwal-Harding KJ, Shearer D (2021). Cost utility analysis of intramedullary nailing and skeletal traction treatment for patients with femoral shaft fractures in Malawi. Acta Orthop Scand..

[27] Zheng DJ, Sur PJ, Ariokot MG, Juillard C, Ajiko MM, Dicker RA (2021). Epidemiology of injured patients in rural Uganda: A prospective trauma registry’s first 1000 days. PLoS One..

[28] Nwanna-Nzewunwa OC, Ajiko M-M, Kirya F (2016). Barriers and facilitators of surgical care in rural Uganda: a mixed methods study. J Surg Res.

[29] Mustafa Diab M, Shearer DW, Kahn JG (2019). The cost of intramedullary nailing versus skeletal traction for treatment of femoral shaft fractures in Malawi: a prospective economic analysis. World J Surg.

[30] Orthopedics Today. Several patient factors may increase length of stay after femur fracture treatment. Orthopedics today. https://www.healio.com/news/orthopedics/20180615/several-patient-factors-may-increase-length-of-stay-after-femur-fracture-treatment#:~:text= Treatment methods included intramedullary nailing,of stay of 6.43 days. Published 2018. Accessed 8 Aug 2018.

[31] Aizpuru M, Staley C, Reisman W, Gottschalk MB, Schenker ML (2018). Determinants of length of stay after operative treatment for femur fractures. J Orthop Trauma..

[32] Maree JE, Cur D, Moshima D, Ngubeni M, Zondi L (2017). On being a caregiver: the experiences of South African family caregivers caring for cancer patients. Eur J Cancer Care (Engl).

[33] Kipp W, Tindyebwa D, Rubaale T (2007). Family Caregivers in Rural Uganda: The Hidden Reality. Health Care Women Int..

[34] Anderson GA, Ilcisin L, Kayima P (2017). Out-of-pocket payment for surgery in Uganda: The rate of impoverishing and catastrophic expenditure at a government hospital. PLoS ONE.

[35] The World Bank (2016). Poverty Headcount Ratio at $1.90 a Day (2011 PPP) (% of Population) - Uganda.

[36] International Food Policy Research Institute (IFPRI) and Datawheel. Soroti, Uganda. https://dataafrica.io/profile/soroti-uga. Published 2017. Accessed 20 Dec 2021.

[37] Devaraj NK (2017). Use of mobile phones to improve follow-up rates. Malawi Med J.

[38] Christie SA, Mbianyor MA, Dissak-Delon FN (2020). Feasibility of a cellular telephone follow-up program after injury in Sub-Saharan Africa. World J Surg.

[39] Kishiki E, Van Dijk K, Courtright P (2016). Strategies to improve follow-up of children after surgery for cataract: findings from child eye health tertiary facilities in sub-Saharan Africa and South Asia. Eye.

