# Supplementary material for: A cost-effectiveness analysis of surgical care delivery in Eastern Uganda-a societal perspective
Source: BMC Health Serv Res. 2023 Mar 15;23:256. doi: 10.1186/s12913-023-09216-x (PMC10015833; doi:10.1186/s12913-023-09216-x)
Supplement: Supplementary file 1 — Additional file 1. [file 12913_2023_9216_MOESM1_ESM.pdf]

**Surname** **First name**

# PATIENT DATA COLLECTION SPREADSHEET

| | | Day 1 | Day 2 | Day 3 | Day 4 | Day 5 | Day 6 | Day 7 | Day 8 | Day 9 | 10 | 11 | 12 | 13 | 14 | 15 | 16 | 17 | 18 | 19 | 20 | 21 | 22 | 23 |
|---|---|---|---|---|---|---|---|---|---|---|---|---|---|---|---|---|---|---|---|---|---|---|---|---|
| | **DAILY QUESTIONS** | | | | | | | | | | | | | | | | | | | | | | | |
| ASK PATIENT | **OCCUPATION** | | | | | | | | | | | | | | | | | | | | | | | |
| ASK PATIENT | Are you able to do your primary work? YES= 1/NO=0 | | | | | | | | | | | | | | | | | | | | | | | |
| ASK PATIENT | If yes, how many hours do you spend on this job | | | | | | | | | | | | | | | | | | | | | | | |
| ASK PATIENT | Other question(s) | | | | | | | | | | | | | | | | | | | | | | | |
| ASK PATIENT | **FAMILY AND SOCIAL HISTORY** | | | | | | | | | | | | | | | | | | | | | | | |
| ASK PATIENT | Number of attendants who visited yesterday | | | | | | | | | | | | | | | | | | | | | | | |
| ASK PATIENT | Number of attendants who spent the night | | | | | | | | | | | | | | | | | | | | | | | |
| | **CLINICAL DATA** | | | | | | | | | | | | | | | | | | | | | | | |
| | **Diagnosis** | | | | | | | | | | | | | | | | | | | | | | | |
| FROM FILE | | | | | | | | | | | | | | | | | | | | | | | | |
| FROM FILE | | | | | | | | | | | | | | | | | | | | | | | | |
| FROM FILE | | | | | | | | | | | | | | | | | | | | | | | | |
| | | | | | | | | | | | | | | | | | | | | | | | | |
| | | | | | | | | | | | | | | | | | | | | | | | | |
| | **Medications** | | | | | | | | | | | | | | | | | | | | | | | |
| | | | | | | | | | | | | | | | | | | | | | | | | |
| FROM FILE | | | | | | | | | | | | | | | | | | | | | | | | |
| FROM FILE | | | | | | | | | | | | | | | | | | | | | | | | |
| FROM FILE | | | | | | | | | | | | | | | | | | | | | | | | |
| FROM FILE | | | | | | | | | | | | | | | | | | | | | | | | |
| FROM FILE | | | | | | | | | | | | | | | | | | | | | | | | |
| FROM FILE | | | | | | | | | | | | | | | | | | | | | | | | |
| FROM FILE | | | | | | | | | | | | | | | | | | | | | | | | |
| FROM FILE | | | | | | | | | | | | | | | | | | | | | | | | |
| FROM FILE | | | | | | | | | | | | | | | | | | | | | | | | |
| FROM FILE | | | | | | | | | | | | | | | | | | | | | | | | |
| FROM FILE | | | | | | | | | | | | | | | | | | | | | | | | |
| FROM FILE | | | | | | | | | | | | | | | | | | | | | | | | |
| FROM FILE | **Procedures (anything done for the patient other than drugs)** | | | | | | | | | | | | | | | | | | | | | | | |
| FROM FILE | | | | | | | | | | | | | | | | | | | | | | | | |
| | | | | | | | | | | | | | | | | | | | | | | | | |
| | | | | | | | | | | | | | | | | | | | | | | | | |
| | | | | | | | | | | | | | | | | | | | | | | | | |
| | | | | | | | | | | | | | | | | | | | | | | | | |
| | | | | | | | | | | | | | | | | | | | | | | | | |
| | | | | | | | | | | | | | | | | | | | | | | | | |
| | | | | | | | | | | | | | | | | | | | | | | | | |
| | | | | | | | | | | | | | | | | | | | | | | | | |
| | **Laboratory tests, CT and other scans** | | | | | | | | | | | | | | | | | | | | | | | |
| | | | | | | | | | | | | | | | | | | | | | | | | |
| | | | | | | | | | | | | | | | | | | | | | | | | |
| | | | | | | | | | | | | | | | | | | | | | | | | |
| FROM FILE | | | | | | | | | | | | | | | | | | | | | | | | |
| FROM FILE | | | | | | | | | | | | | | | | | | | | | | | | |
| FROM FILE | | | | | | | | | | | | | | | | | | | | | | | | |
| FROM FILE | | | | | | | | | | | | | | | | | | | | | | | | |
| FROM FILE | | | | | | | | | | | | | | | | | | | | | | | | |
| FROM FILE | | | | | | | | | | | | | | | | | | | | | | | | |
| | | | | | | | | | | | | | | | | | | | | | | | | |
| | **RESOURCE UTILIZATION** | | | | | | | | | | | | | | | | | | | | | | | |
| | **Where did you sleep last night? (YES=1, NO= 0)** | | | | | | | | | | | | | | | | | | | | | | | |
| ASK PATIENT | **Complications** (specify if preop/ due to surgery) | | | | | | | | | | | | | | | | | | | | | | | |
| ASK PATIENT | | | | | | | | | | | | | | | | | | | | | | | | |
| ASK PATIENT | | | | | | | | | | | | | | | | | | | | | | | | |
| ASK PATIENT | | | | | | | | | | | | | | | | | | | | | | | | |
| ASK PATIENT | | | | | | | | | | | | | | | | | | | | | | | | |
| ASK PATIENT | | | | | | | | | | | | | | | | | | | | | | | | |
| ASK PATIENT | | | | | | | | | | | | | | | | | | | | | | | | |
| ASK PATIENT | **RESOURCE UTILIZATION** | | | | | | | | | | | | | | | | | | | | | | | |
| ASK PATIENT | **Where did you sleep last night? (YES=1, NO= 0)** | | | | | | | | | | | | | | | | | | | | | | | |
| ASK PATIENT | 1. Private ward | | | | | | | | | | | | | | | | | | | | | | | |
| ASK PATIENT | 2. On a bed alone in the general ward | | | | | | | | | | | | | | | | | | | | | | | |
| ASK PATIENT | 3. Shared a bed | | | | | | | | | | | | | | | | | | | | | | | |
| ASK PATIENT | 4. On the floor in the ward | | | | | | | | | | | | | | | | | | | | | | | |
| ASK PATIENT | 5. In a storage room/ other room | | | | | | | | | | | | | | | | | | | | | | | |
| ASK PATIENT | 6. Outside the ward (in the open) | | | | | | | | | | | | | | | | | | | | | | | |
| ASK PATIENT | | | | | | | | | | | | | | | | | | | | | | | | |
| ASK PATIENT | **How much water did you use yesterday while at SRRH?** | | | | | | | | | | | | | | | | | | | | | | | |
| ASK PATIENT | 1. Buckets | | | | | | | | | | | | | | | | | | | | | | | |
| ASK PATIENT | 2. Bottles | | | | | | | | | | | | | | | | | | | | | | | |
| ASK PATIENT | | | | | | | | | | | | | | | | | | | | | | | | |
| ASK PATIENT | **Were you able to use the toilet in the hospital yesterday? (YES=1, NO=0, N/A= Not applicable)** | | | | | | | | | | | | | | | | | | | | | | | |
| ASK PATIENT | **If No/ Not applicable** | | | | | | | | | | | | | | | | | | | | | | | |
| ASK PATIENT | 1. Why? | | | | | | | | | | | | | | | | | | | | | | | |
| ASK PATIENT | 2. What alternative did you use? | | | | | | | | | | | | | | | | | | | | | | | |
| ASK PATIENT | | | | | | | | | | | | | | | | | | | | | | | | |
| | **SURGICAL WORKFORCE UTILIZATION** | | | | | | | | | | | | | | | | | | | | | | | |
| YOU OBSERVE | How much time did the surgical team spend on the patient during rounds today? | | | | | | | | | | | | | | | | | | | | | | | |
| YOU OBSERVE | | | | | | | | | | | | | | | | | | | | | | | | |
| JMBER OF PEOPLE | How many of the following health care providers were at the rounds? | | | | | | | | | | | | | | | | | | | | | | | |
| YOU OBSERVE | 1. Consultant surgeons | | | | | | | | | | | | | | | | | | | | | | | |
| YOU OBSERVE | 2. Medical officers | | | | | | | | | | | | | | | | | | | | | | | |
| YOU OBSERVE | 3. Intern doctors | | | | | | | | | | | | | | | | | | | | | | | |
| YOU OBSERVE | 4. Orthopedic officers | | | | | | | | | | | | | | | | | | | | | | | |
| YOU OBSERVE | 5. Nurses | | | | | | | | | | | | | | | | | | | | | | | |
| | 6. Anaesthetist nurse | | | | | | | | | | | | | | | | | | | | | | | |
| IME SPENT (MINS) | How many of the following cadres of providers were at the rounds? | | | | | | | | | | | | | | | | | | | | | | | |
| YOU OBSERVE | 1. Consultant surgeons | | | | | | | | | | | | | | | | | | | | | | | |
| YOU OBSERVE | 2. Medical officers | | | | | | | | | | | | | | | | | | | | | | | |
| YOU OBSERVE | 3. Intern doctors | | | | | | | | | | | | | | | | | | | | | | | |
| YOU OBSERVE | 4. Orthopedic officers | | | | | | | | | | | | | | | | | | | | | | | |
| YOU OBSERVE | 5. Nurses | | | | | | | | | | | | | | | | | | | | | | | |
| YOU OBSERVE | 6. Other (specify) | | | | | | | | | | | | | | | | | | | | | | | |
| | **How many** of the following **health care providers** were **DURING SURGERY** | | | | | | | | | | | | | | | | | | | | | | | |
| | 1. Consultant surgeons | | | | | | | | | | | | | | | | | | | | | | | |
| | 2. Medical officers | | | | | | | | | | | | | | | | | | | | | | | |
| | 3. Intern doctors | | | | | | | | | | | | | | | | | | | | | | | |
| | 4. Orthopedic officers | | | | | | | | | | | | | | | | | | | | | | | |
| | 5. Nurses | | | | | | | | | | | | | | | | | | | | | | | |
| | 6. Anaesthetist nurse | | | | | | | | | | | | | | | | | | | | | | | |
| | | | | | | | | | | | | | | | | | | | | | | | | |
| | **How much time** did they spend **IN SURGERY (min)?** | | | | | | | | | | | | | | | | | | | | | | | |

**Surname** **First name**

# PATIENT DATA COLLECTION SPREADSHEET

| PATIENT ENROLLMENT QUESTIONS | |
|---|---|
| **DEMOGRAPHIC DATA** | |
| Study subject number | LOS/PX/ |
| Age | |
| Sex | |
| Marital status | |
| Address | |
| Village | |
| District | |
| Highest level of education | |
| Primary occupation | |
| How many hours a day do you spend on this job | |
| Primary source of income | |
| **TRANSPORTATION TO HOSPITAL** | **Cost** |
| Bus | UGX - |
| Taxi | UGX - |
| Ambulance | UGX - |
| Police car | UGX - |
| Bicycle | UGX - |
| Boda boda (motorcycle) | UGX - |
| Personal vehicle/ friend | UGX - |
| Total transportation cost to hospital | UGX - |
| **[illegible]MILY AND SOCIAL HISTORY** | |
| Position/ role in immediate family | |
| Number of dependants | |
| | |
| | |
| **CLINICAL DATA** | |
| Date of Admission | |
| | |
| **ASSESSMENT SOCIOECONOMIC STATUS** | |
| Phone number | |
| Phone number | |
| | |
| | |
| | |
| **EQ 5D disability assessment atarrival (see pdf)** | Range = 1 - 5 |
| Mobility | |
| Self-care | |
| Usual activities (work, study, housework, family activities) | |
| Pain/ discomfort | |
| Anxiety/ depression | |
| **[illegible]isual analogue scale** | Range 1-100 |
| [illegible]elf-assessment of health using health scale (0-100) | |

| PATIENT DISCHARGE QUESTIONS | | |
|---|---|---|
| **CLINICAL DATA** | | |
| Date of discharge | | |
| **REASON FOR DISCHARGE?** | | |
| | | |
| **Condition** | **Intervention received** | **Status: Resolved? Yes/No?** |
| Diagnosis | | |
| Diagnosis 2 (include name here) | | |
| Diagnosis 3 (include name here) | | |
| | | |
| **EQ 5D disability assessment at DISCHARGE (se** | Range = 1 - 5 | **Total EQ 5D score** |
| Mobility | | 0 |
| Self-care | | |
| Usual activities (work, study, housework, family activities) | | |
| Pain/ discomfort | | |
| Anxiety/ depression | | |
| **Visual analogue scale** | | |
| Self-assessment of health using health scale (0-100) | | |
| **TRANSPORTATION HOME** | | |
| Mode of transportation and cost | Estimated cost | |
| Bus | UGX - | |
| Taxi | UGX - | |
| Ambulance | UGX - | |
| Police car | UGX - | |
| Boda boda (motorcycle) | UGX - | |
| Personal vehicle/ friend | UGX - | |
| Total transportation cost to hospital | UGX - | |
| | | |
| **RESOURCE UTILIZATION** | | |
| AT SRRH, what do you use electricity for mostly? | | |
| 1. Charging your phone | | |
| 2. Charging your torch | | |
| 3. Radio | | |
| 4. Other (please specify) | | |
| hospital? | | |
| If you cook, where do they get the cooking fuel? | | |

| OPEN-ENDED QUESTIONS AT DISCHARGE | |
|---|---|
| How has this hospital admission affected your family or life? | |
| How has this hospital admission affected your job? | |
| How has this hospital admission affected your finances? | |
| Did you or your family or relatives sell any property or make any hard sacrifices in order for you to access care? | |
