# Supplementary material for: A cost-effectiveness analysis of surgical care delivery in Eastern Uganda-a societal perspective
Source: BMC Health Serv Res. 2023 Mar 15;23:256. doi: 10.1186/s12913-023-09216-x (PMC10015833; doi:10.1186/s12913-023-09216-x)
Supplement: Supplementary file 2 — Additional file 2. [file 12913_2023_9216_MOESM2_ESM.pdf]

Surname **First name**

ATTENDANT'S DATA COLLECTION SPREADSHEET

ATTENDANT ENROLLMENT QUESTIONS	
**DEMOGRAPHIC DATA**	
Attendant study number	*ATT/LOS/PX...../ ......*
Age	
Sex	
Marital status	
Address	
Village	
District	
Primary occupation	
Primary source of income	
Highest level of education	
Mode of transportation and cost	Cost
Bus	
Taxi	
Ambulance	
Police car	
Boda boda (motorcycle)	
Personal vehicle/ friend	
Total transportation cost to hospital	UGX -
Where did you travel from?	
On what date did you arrive SRRH on this your current visit?	
How long did it take you to get to the hospital on this visit? (Mins)	
**VISITATION DATA**	
Date of first visit	01/01/17
Date of discharge	
**FAMILY AND SOCIAL HISTORY**	
What is your relationship with the patient?	
What is your position/ role in your immediate family?	
How many people are dependent on you?	
Other pertinent information (please specify)	
**RESOURCE UTILIZATION**	
AT SRRH, what do you use electricity for mostly?	
1. Charging your phone	
2. Charging your torch	
3. Radio	
4. Other (please specify)	
Do you **BUY** food or do you **COOK** it in the hospital?	
If you cook, where do they get the cooking fuel?	
Phone number	

ATTENDANT DISCHARGE QUESTIONS	
**MODE OF TRANSPORTATION**	
Last day of visit/ stay	01/01/17
Mode of transportation and cost	Cost
Bus	
Taxi	
Ambulance	
Police car	
Boda boda (motorcycle)	
Personal vehicle/ friend	
Total transportation cost to hospital	UGX -

DAILY QUESTIONS	
**OCCUPATION**	
Are you able to do your primary work? YES/NO	
Other question(s)	
**RESOURCE UTILIZATION**	
**Where did you sleep last night? (YES=1, NO= 0, 98= Not applicable)**	
At home (not in the hospital)	
On a bed alone in the ward	
Shared a bed	
On the floor in the ward	
In a storage room/ other room	
Outside the ward (in the open)	
**How much water did you use yesterday while at SRRH?**	
Buckets	
Bottles	
**Did you use the toilet in the hospital yesterday? (YES=1, NO=0, N/A= Not applicable)**	
**If No/ Not applicable**	
Why?	
What alternative did you use?	
**VISITATION DATA**	
**Arrival**	
FILL IF ATTENDANT COMES AND GOES	
FILL IF ATT Mode of transportation and cost	
FILL IF ATT Bus	
FILL IF ATT Taxi	
FILL IF ATT Ambulance	
FILL IF ATT Police car	
FILL IF ATT Boda boda (motorcycle)	
FILL IF ATT Walking	
FILL IF ATT Personal vehicle/ friend	
FILL IF ATT How much did it cost you to transport yourself to SRRH	
FILL IF ATTENDANT COMES AND GOES	
FILL IF ATT **Departure**	
FILL IF ATT Where are you going to	
FILL IF ATT Mode of transportation and cost	
FILL IF ATT Bus	
FILL IF ATT Taxi	
FILL IF ATT Ambulance	
FILL IF ATT Police car	
FILL IF ATT Boda boda (motorcycle)	
FILL IF ATT Walking	
FILL IF ATT Personal vehicle/ friend	
FILL IF ATT Cost of transportation to SRRH	
FILL IF ATT How much will/ did it cost you to transport yourself home?	

How has this hospital admission affected your family or life?	
How has this hospital admission affected your job?	
How has this hospital admission affected your finances?	
Did you or your family or relatives sell any property or make any hard sacrifices in order for you to access care?	
